# Implementation of national guidelines for the prevention and treatment of overweight and obesity in children and adolescents: a phenomenographic analysis of public health nurses’ perceptions

**DOI:** 10.3402/qhw.v11.31934

**Published:** 2016-08-18

**Authors:** Aina Nordstrand, Bengt Fridlund, Ragnhild Sollesnes

**Affiliations:** 1 Barn og unge tjenesten, Alta, Norway; 2 School of Health and Welfare, Jönköping University, Jönköpig, Sweden; 3 Faculty of Health and Social Sciences, Bergen University College, Bergen, Norway

**Keywords:** Evidence-based nursing, health promotion, risk factors, qualitative methods, school health service, well-baby clinic

## Abstract

**Objective:**

To explore and describe how public health nurses (PHNs) perceive the implementation of national guidelines for the prevention and treatment of overweight and obesity among children and adolescents in well-baby clinics and school health services.

**Design, sample, and measurements:**

An explorative descriptive design was carried out through individual interviews with 18 PHNs and analysed according to the phenomenographic tradition.

**Results:**

Four implementation strategies were described and assigned a metaphor: the structured PHN, pragmatic PHN, critical PHN, and the resigned PHN. Competence, patient receptiveness, internal consensus, interdisciplinary collaboration, resources, and organizational embedding were the determinants identified that most frequently affect implementation, and these determinants were distributed at different levels of the organization. The extent of facilitation seemed to determine which implementation strategy would be used.

**Conclusions:**

How PHNs implemented the guidelines for overweight and obesity were affected by determinants at different organizational levels. Contextual facilitation of implementation seemed better in larger organizations, but factors such as leadership, drive, and experience compensated in smaller municipalities. The implementation of guidelines was hindered when the barriers exceeded the benefits.

Overweight and obesity in children is a worldwide challenge (de Onis, Blossner, & Borghi, 2010; Wijnhoven et al., 2013). In Norway, around 14% of children aged 2–19 years are overweight and approximately 2% of the same age group are obese (Juliusson et al., 2010). In 2010, the Norwegian Directorate of Health released national guidelines for the prevention and treatment of overweight and obesity in children and adolescents, targeting primary healthcare (Helsedirektoratet, 2010). Norwegian public health nurses (PHNs), who work in the areas of health promotion and primary healthcare, are recommended to act on both structural and individual levels to prevent the development of overweight. They also have to contribute to prevent and reduce obesity among children and adolescents. According to Glavin, Schaffer, Halvorsrud, and Kvarme (2014), Norwegian PHNs find that responding to overweight and obesity requires evidence to provide the best healthcare. Evidence-based practice combines professional expertise, the most relevant research, and patient preferences and values within a specific context (Melnyk & Fineout-Overholt, 2011). Evidence-based nursing is recommended for PHNs (Brownson, Fielding, & Maylahn, 2009), and clinical guidelines form part of the toolkits that have been developed to make knowledge more accessible to public health services and PHNs (Kelly et al., 2010).

Implementation science investigates agile ways to integrate research findings and evidence, and it is generally agreed that the implementation of guidelines is a challenging and complex task (Fixsen, Blase, Naoom, & Wallace, 2009; Graham et al., 2006; Titler, Everett, & Adams, 2007). Implementation can be described as the introduction of new scientific insight, with the aim that it be given a structural place in practice (Grol & Wensing, 2013a). It is well documented that the implementation of an innovation is affected by several factors (Wandersman et al., 2008). According to Grol and Wensing's (2013b) model, it has been concluded that these comprise the actual innovation; the practitioners; the clients; culture in the workplace; the economic, administrative, and organizational context; and the choice of strategy for the implementation and dissemination of the innovation. Accordingly, a Swedish study showed that guideline developers could benefit from an initial assessment of how the actual topic, the target context, and the stakeholders affected the implementation (Richter-Sundberg, Kardakis, Weinehall, Garvare, & Nystrom, 2015). In addition, a Canadian study found several barriers to the implementation of best practice guidelines into a public health setting, which were consistent with earlier research, such as time constraints, working in multidisciplinary teams, and system-level changes in leadership (Athwal et al., 2014). In Norway, the use of research by PHNs during consultations concerning childhood vaccination was investigated, and national guidelines proved to be important sources of information for these healthcare providers (Austvoll-Dahlgren & Helseth, 2010). Yet, there is a lack of evidence about the impact of nursing best practice guidelines and the most effective strategies for the implementation of these guidelines (Davies, Edwards, Ploeg, & Virani, 2008). Taking this into account, it is important to learn more about how PHNs implement national guidelines into their practice. To our knowledge, no study to date has focused on implementation in this way; therefore, the aim of this study was to explore and describe how PHNs perceive the implementation of a national guideline directed towards overweight and obesity among children and adolescents.

## Materials and methods

### Design and method description

To gain insight into how PHNs perceive the implementation of a national guideline, an explorative design with a phenomenographic approach was chosen as it aims to describe and understand *how* the world is perceived by people. Phenomenography was developed in the 1970s within educational research (Marton, 1970), but it has been used in healthcare research since the 1990s (Sjöström & Dahlgren, 2002). In phenomenography, a distinction is made between the world as it is and the world as it is perceived by people. The former perspective is labelled the first-order perspective (the what), and the latter, the target of a phenomenographic study, is labelled the second-order perspective (the how) (Marton, 1981). People perceive phenomena in different ways; however, the process of creating meaning is limited, and studies have shown that there are between two and six qualitatively different ways of perceiving the same phenomenon. The categories of description are the main outcome of a phenomenographic study. These represent possible ways of perceiving the phenomena and express the researcher's interpretation of what has been described by the participants. It is important to emphasize that the categories of description refer to the collective level and not to individuals, but each participant can express a dominant as well as a non-dominant perception of the phenomenon searched for (Larsson & Holmström, 2007).

### Context

PHNs in Norway work in school health services, youth health clinics, and well-baby clinics in municipalities (Glavin et al., 2014). These services are organized differently, but PHNs work under the same regulations and guidelines throughout the country (Forskrift om helsestasjons-og skolehelsetjenesten, 2003). PHNs may be collocated with other services; some may work in facilities for educational and psychological services, child welfare and habilitation, whereas others may share premises with physicians, midwives, and physiotherapists.

### Study participants

The PHNs in this study all worked in school health service facilities or well-baby clinics. All participants had completed a baccalaureate nursing programme and had obtained public health nursing certification (Glavin et al., 2014). The sample represented various regions as well as different sized municipalities (Table I). All PHNs were engaged in the implementation of guidelines as either a leader, a project member, part of an interdisciplinary group, or because they had a special interest in the topic. An inclusion criterion, and one that emerged as crucial, was that PHNs had enough time to participate in an interview lasting 30–60 min. In all, 31 PHNs were invited to participate by telephone or mail. Three PHNs declined to participate, and 10 never responded, leaving 18 PHNs who participated in the study.

**Table I:** Sociodemographic and clinical characteristics of public health nurses (*n*=18).

	No.
Sex	
Female	18
Age (years)	
<40	1
40–49	10
50–60	6
>60	1
Professional position	
Public health nurse	10
Other (leader, nurse practitioner, project member)	8
Years as public health nurse	
<6	3
6–10	5
11–15	6
16–20	2
>20	2
Municipality size	
1000–4999	4
5000–9999	4
10,000–29,999	3
30,000–99,999	3
100,000–200,000	2
>200,000	2
Area	
Northern Norway	8
Central Norway	2
Western Norway	4
Eastern Norway	3
Southern Norway	1

### Data collection

Except for one interview conducted at a PHN's office, all interviews were conducted by telephone and audiotaped by the first author, between October 2013 and April 2014. The interviewer (also a PHN) and participant each sat in an undisturbed room for the interview. Initially, each participant was informed about the aim of the study and legal regulations related to collecting sensitive information. An interview guide compiled by the authors, who are familiar with the method and topic, contained an open-ended question and two additional questions to reveal the concrete experiences of PHNs (Larsson & Holmström, 2007) and keywords from implementation theory (Bahtsevani, Willman, Stoltz, & Ostman, 2010; Grol & Wensing, 2013b; Spyridonidis & Calnan, 2010). The main question was “The national guidelines for prevention and treatment of overweight and obesity in children and adolescents were enacted in 2010. How do you experience implementation of the guidelines in the school health services and well-baby clinics in your district?” At the same time, participants were asked to keep in mind two other questions: “Have you experienced any barriers to guideline implementation?” and “Have you experienced any incentives to their implementation?” Probing questions were asked, such as “Can you tell me more about that?” Each interview lasted 27–70 min, with a median of 49 min. A pilot interview was conducted and included in the study as no revision of the questions was needed, and the answers were pertinent and comprehensive.

### Ethical considerations

The Norwegian Social Science Data Archive (Norsk Samfunnsvitenskapelig Datatjeneste) approved the study (Project No. 34793). Participants received a letter with information about the study and about confidentiality, which also stated that they could withdraw their consent at any time.

### Data analysis

The first author transcribed each interview verbatim soon after it was concluded. The analysis was carried out according to the procedure of Larsson and Holmström (2007). Determining the correct perspective was ensured by reading each interview transcript twice, so as to extract answers to the main questions while looking for both “the what” and “the how.” The phenomenographic approach is concerned not only with what participants are saying but also with how they express themselves, that is, the underlying meanings. Preliminary descriptions of the predominant ways that PHNs experienced implementation of the guidelines were compiled. Essentially no new descriptions were given after the 11th interview. Descriptions were grouped based on what was perceived to be similarities and differences. This part of the analysis was demanding because the text (the what) had to be kept independent from the experiences (the how). There were 118 descriptions in total, and Table II indicates to which interview each statement was connected. All authors had access to the data and were involved in the analysis process at all levels, that is, discussions and reflections were essential until negotiated consensus (Dahlgren & Fallsberg, 1991) could be reached. Categories of description emerged and were each assigned a metaphor. Transcripts were reread to look for non-dominant perceptions. The categories of description and their internal structure constituted the outcome space.

**Table II:** Overview of phenomenographic analysis with regard to categories, statements, and participating public health nurses (*n*=18).

Categories of description and perceptions	No. of statements	No. of participants
Structured PHN		
• Ensured interdisciplinary cooperation	78	1–14, 16–18
• Integrated new practice into routines	74	1–18
• Planned and evaluated the implementation	59	1–18
• Ensured sufficient competence	25	1–10, 13–16, 18
Pragmatic PHN		
• Adjusted implementation to the existing competence	83	1–18
• Implemented when PHNs agreed to do so	33	1–15, 17–18
• Adjusted the implementation to maintain patient autonomy	27	1–2, 5–12, 14, 16–18
• Implemented regardless of organizational embedding	8	1–3, 7, 13, 17–18
Critical PHN		
• Did not implement owing to resistance from leadership	70	1–18
• Did not implement owing to lack of resources	41	1–12, 14 –18
• Did not implement because PHN considered it unethical	35	1–10, 13–18
• Did not implement because PHNs agreed not to do so	16	1–2, 4–9, 11–18
Resigned PHN		
• Did not implement owing to lack of organizational support	39	2–4, 6–7, 9–12, 14–17
• Did not implement owing to lack of resources	24	1–2, 4–8, 10–14, 16–18
• Did not implement because other health practitioners were unsupportive or unavailable	10	11, 13–14, 17
• Did not implement because families were unreceptive	8	2, 11, 14, 16, 18

PHN: public health nurse

## Results

The metaphors that emerged represented variations in the perceptions of PHNs regarding implementation of the national guidelines at a descriptive level. We did not investigate who the PHNs were but rather how they expressed themselves, meaning that the perceptions were detached from the person expressing them. The metaphors were as follows: “the structured PHN,” “the pragmatic PHN,” “the critical PHN,” and “the resigned PHN.” Perceptions connected to each metaphor are listed in Table II.

### The structured PHN

This metaphor refers to PHNs who were aware of the new guidelines at an early stage. These PHNs believed that the changes led to better nursing for families and ensured that all staff had been familiarized with the guidelines. These PHNs adopted the guidelines to the system by integrating them into their normal routines. Quality assurance was important to these PHNs: “We didn't know if this was a good way to do it. We didn't know if this was the final way. We wanted to make sure there was room to improve, and we wanted everyone to focus on the quality assurance this would lead to.” Structured PHNs had everything in order: the budget, competence, and cooperation with other healthcare professionals relevant to the guidelines. These PHNs strived to overcome barriers to change both within the organization and among their colleagues. Despite barriers, the PHNs remained loyal to the guidelines:

And we don't have to agree with it. I sometimes ask myself will this lead to improving health? I keep wondering… It is important that we do what is expected, but not at any cost. We offer; we follow the guidelines, we do the documentation that is expected, but we reflect a little: is this working out well?

New routines were based on internal reflections among PHNs, training days, peer teaching, and also external courses when needed. Structured PHNs cooperated with other healthcare professionals to tailor interventions to suit the kind of challenges faced by the families they worked with. Physiotherapists, physicians, and dieticians were often mentioned. Structured PHNs were supported in implementation of the guidelines by organizational determinants. “The city council and the politicians support the work. They realize this is innovative and has economic implications, which are positive in the long run.” These PHNs were given the influence to be able to implement the guidelines by their position as a leader, as a nurse practitioner, working on a project, or because of a special interest in the topic. These PHNs carried out the assignment in a structured way, loyal to the system, and considered implementation to be important to quality improvement.

### The pragmatic PHN

This metaphor corresponded to PHNs who considered the guidelines useful for their work with children with overweight and obesity and their families. They became familiar with the guidelines and adopted new methods as these PHNs found them relevant to their practice. Pragmatic PHNs considered implementation to be a process. These PHNs sought out people in their municipality who possessed useful skills or positions, to help them make the changes required. Pragmatic PHNs considered the perspectives of the families and were always concerned with families being offered the best possible services:

Yes, first of all you want to do it in a respectful manner, because many of the parents feel they have failed when they see the percentile pointing in the wrong direction. Luckily, we've achieved a good dialogue and a good atmosphere with most of the families. But we've been thinking and reflecting a lot on which methods to use to motivate the parents, and also to explain…. I think these guidelines are so useful in that way. The questions you need to ask the parents, and the questions you need to ask the adolescents, are all suggested.

Pragmatic PHNs began implementing national guidelines in the school health services and well-baby clinics based on their own experience and the internal reflections of PHNs, adjusting to resources within the organization: “…we simply had to find our capacities, what is possible for us to perform.” The pragmatic PHNs considered organizational support positive and took the opportunity to influence people in power whenever possible: “… we don't intermingle, don't have lunch together, don't see each other. I think having the decision makers nearby matters too, in all this.” These PHNs were determined and engaged, always looking for opportunities. They did not consider implementation of the guidelines dependent on cooperation with other healthcare professionals; however, if this seemed possible, pragmatic PHNs would initiate it.

### The critical PHN

Critical PHNs knew the guidelines well and considered them useful to PHNs and families. At the same time, these PHNs made it clear that that they felt it unethical to implement the new guidelines if the only intervention was a conversation about health at the PHN's office:

I remember how ambivalent I was when the guidelines came. I already felt that we didn't have enough time to accomplish all our tasks, and all the overweight that was revealed … at the same time I considered it an important issue, because I noticed that some children are heavier than what is good for them, and some are underweight too. But it does no good to the children and adolescents to know their numbers, as long as we have nothing to offer them.

The guidelines’ target group is primary care. Critical PHNs stated that they would not implement the guidelines unless physicians, physiotherapists, dieticians, and others assumed their part of the responsibility and unless necessary interventions were established:

We don't agree with the recommendations putting such a lot of responsibility on the PHNs. In our opinion, to implement the guidelines, we need more resources, more cooperation, and for now, the regular GPs must take more responsibility.

Before the guidelines were introduced, these PHNs already faced priorities that threatened their professional credibility. They did not implement anything unless there were a sufficient number of PHNs available at work, thus making it possible to develop new routines and interventions according to the guidelines and without having to neglect any other tasks:

Yes, we started, but then we realized, also at the request of the Interest group of public health nurses, that with this amount of resources, is it possible to do a qualitatively good job? In our opinion it's necessary to increase the budget, making it possible for us to offer the families the good nursing they deserve. The topic is quite demanding, affecting feelings and interaction in the families, and we figured – status quo, we cannot do it.

Critical PHNs considered follow-up by the PHNs to be worthless unless families realized the need for change. This was important to them because these PHNs felt suffocated by the priorities they had to make relative to other important tasks. Critical PHNs presupposed that their municipality would facilitate implementation of the guidelines. These PHNs wished to implement the guidelines and were ready to do so, but not to do so alone. They wanted the guidelines to be embedded within the organization such that a sufficient number of incentives provided PHNs with the space they needed to implement the guidelines with good quality.

### The resigned PHN

The perceptions of resigned PHNs were that the guidelines covered an area with potential for improvement. Because these PHNs quite often worked alone, however, they found the guidelines overwhelming and demanding: “I think I found them overwhelming. We were wondering how on earth we could get the job done. We didn't have what was needed; the municipality didn't have what it takes to work on this. So yes, they were quite overwhelming.” Resigned PHNs wanted the guidelines to contain more documented interventions and more concrete tools that were ready to use:

And I would have wished for, as the Norwegian Directorate of Health published guidelines like these, that a “package” would follow: tested, quality assured interventions with available external courses listed and so on. I think that would have made the process so much easier for municipalities in Norway.

The fact that municipalities did not facilitate implementation to a great extent made it clear to the resigned PHNs that they could not begin to implement the guidelines. The challenges these PHNs faced were about infrastructure and non-urban factors, such as few people living in large areas and healthcare professionals that were spread out across large distances, with both of these groups out of the reach of PHNs to easily manage or influence. The system was less robust owing to few meeting points, few incentives for interdisciplinary activities, and vacancies and frequent replacement of key personnel. Resigned PHNs worked in positions with many different functions. This made it difficult for them to set priorities and keep their professional integrity intact. The fact that the guidelines were not embedded at other organizational levels made implementation in well-baby clinics and school health services seem an even more challenging and lonely task to these PHNs:

And even if they're sent to the counsellor or other local authorities, they just forward them down the system and forget they ever existed. And if you have the time or capacity to familiarize yourself with them, then you realize this means an awful lot of work. How can I say this – there is so much work to do and not only for the well-baby clinic or the school health service – and sometimes it feels like – it seems like no one else in the municipality contributes.

Resigned PHNs had a good overview of the child population and worked well together with families at an individual level. But as a consequence of the rural factors mentioned above, working at group level was challenging to these PHNs. It could be difficult to gather children with overweight or obesity in a group because of great distances or because of parental concern about stigma in small, transparent societies.

## Discussion

This qualitative analysis using a phenomenographic approach provided more knowledge of the various ways in which PHNs perceive implementation of a national guideline. The different categories of description turned out to be different strategies for handling implementation. Some determinants seemed to more strongly affect implementation: competence, receptiveness among children and families, internal consensus, interdisciplinary collaboration, resources, and organizational embedding. These determinants correspond substantially to the central factors that most individuals and organizations face when implementing new knowledge to change practice, according to the model of Grol and Wensing (2004) (Table III).

**Table III:** Determinants identified that affected implementation of a national guideline in PHNs’ practice; adapted from Grol and Wensing (2004).

Level	Barriers or incentives	Determinants identified
Innovation	Advantages in practice, feasibility, credibility, accessibility, attractiveness	
Individual professional	Awareness, knowledge, attitude, motivation to change, behavioural routines	Competence
Patient	Knowledge, skills, attitudes, compliance	Receptiveness among children and families
Social context	Opinions of colleagues, culture of the network, collaboration, leadership	Internal consensus, interdisciplinary collaboration
Organizational context	Organization of care processes, staff, capacities, resources, structures	Resources, organizational embedding
Economic and political context	Financial arrangements, regulations, policies	

### Competence

Working to prevent overweight and obesity requires a certain competence (Helse- og omsorgsdepartementet, 2013; Leeman et al., 2014). Structured PHNs ensured that all PHNs were competent; they were offered courses, and peer teaching and internal reflections were facilitated. Large, mature, and differentiated organizations, especially those focused on professional knowledge, assimilate innovations more readily (Greenhalgh, Robert, MacFarlane, Bate, & Kyriakidou, 2004). Pragmatic PHNs considered the guidelines a useful source of knowledge. They based implementation on the competence that already existed among PHNs. Professional expertise plays an important role in evidence-based practice, as there are several dimensions for PHNs to consider before deciding what type of care to provide. Implementation flows more easily when there is an ability to link new knowledge to existing knowledge and then put it into practice (Greenhalgh et al., 2004). Critical PHNs called for more concrete answers in the guidelines. Critical thinking in nursing is part of the process when implementing guidelines, and evidence-based practice includes a degree of certainty that the action will lead to a positive impact on patient health (Athwal et al., 2014; Melnyk & Fineout-Overholt, 2011). Resigned PHNs were not very familiar with the new guidelines; therefore, they worked with child overweight and obesity in the same way as before the guidelines were established. PHNs in Norway feel confident in national guidelines (Austvoll-Dahlgren & Helseth, 2012). Possessing relevant competence about overweight seems to make implementation flow more easily.

### Receptiveness of children and families

Overall, the PHNs we interviewed expressed motivation to provide children and their families with quality nursing. Structured PHNs considered guideline implementation a type of quality assurance that would lead to more knowledge among families and better cooperation between PHNs and families. When nurses find that guidelines improve quality and are a useful tool in practice, the guidelines are more likely to be implemented (Bahtsevani et al., 2010; Ploeg, Davies, Edwards, Gifford, & Miller, 2007). Pragmatic PHNs ensured that implementation was tailored to suit the challenges of families. They facilitated the empowering of families and aimed to underpin their ability to make healthy choices. Critical PHNs expected families themselves to understand the need for change. Because this was not the experience of these PHNs, this meant a barrier to implementation. According to research, patients resist recommendations because they believe they do not need or they feel threatened by such help (Cabana et al., 1999). Resigned PHNs experienced patient resistance as a barrier to implementation. These PHNs referred to unreceptive families as “invisible” because they did not understand for themselves the need for change. Patient preferences are part of the evidence and if these are positive, implementation is facilitated (Rycroft-Malone et al., 2002, 2004). How PHNs experienced patient receptiveness seemed to affect their will to implement the guidelines.

### Internal consensus

Structured PHNs identified resistance among their colleagues and ensured that all staff had sufficient knowledge and organizational support to maintain adherence to the guidelines. Pragmatic PHNs emphasized agreement, drive, and exploiting opinion; they were leaders owing to their ability to inspire others (Greenhalgh et al., 2004; Grol & Wensing, 2013b). Implementation strategies that promote personal ownership are more likely to succeed (Monsen et al., 2015). Critical PHNs disagreed with guideline implementation because the number of barriers exceeded the desired effects. There is evidence that new knowledge is more easily adopted when the need for it is identified in practice, a bottom-up instead of a top-down approach (Bahtsevani et al., 2010). Resigned PHNs experienced a lack of meeting points, which probably affected the climate for change by acting as a barrier to internal reflection, a prerequisite for consensus, and a symbol of limited absorptive capacity when absent (Greenhalgh et al., 2004). Lack of agreement has been found to be a barrier to adherence to guidelines (Helse- og omsorgsdepartementet, 2013). The perceptions expressed in this study show that different determinants affect the degree of internal consensus among PHNs, and identifying these in advance is likely to promote implementation.

### Interdisciplinary cooperation

The PHNs in this study considered interdisciplinary cooperation to be an important part of implementation. A Swedish study found that interdisciplinary cooperation led to more knowledge and thus greater confidence in implementation and a consensus to implement (Bahtsevani et al., 2010). However, implementers must be aware that knowledge might differ between professional groups (Kardakis, Weinehall, Jerdén, Nyström, & Johansson, 2014). Structured PHNs primarily worked in large organizations with access to other healthcare professionals and experts. Interdisciplinary cooperation was structured and integrated into daily routines, which increased the likelihood of success (Greenhalgh et al., 2004). Pragmatic PHNs primarily worked in organizations that supported interdisciplinary cooperation, flexibility, and creativity. Attitude and intention to cooperate facilitate the implementation of innovations in nursing (Greenhalgh et al., 2004; Monsen et al., 2015; Ploeg et al., 2007). Pragmatic PHNs were dedicated, involving others by motivating and initiating cooperation. Being able to connect with other organizations in the community is part of a general capacity to implement change within organizations (Wandersman et al., 2008). Critical PHNs experienced that other healthcare professionals were unaware of new guidelines, one reason why interdisciplinary cooperation was difficult to establish. A lack of interventions tailored to suit challenges in the guidelines was seen as a barrier among these PHNs. There is a lack of receptiveness within organizations and across disciplines that hinders the implementation of guidelines (Greenhalgh et al., 2004). Barriers within an organization that are out of the control of healthcare professionals affect the implementation of innovations (Greenhalgh et al., 2004). Resigned PHNs were surrounded by such barriers, such as organizational structures and infrastructures that were incompatible with interdisciplinary cooperation. Considering the complexity of a guideline, a structured plan is recommended (Kajermo et al., 2010). Establishing structures within organizations that facilitate interdisciplinary work should be part of any plan for implementing interdisciplinary-oriented guidelines.

### Resources

The PHNs we interviewed expressed that implementing the national guidelines in school health services and well-baby clinics required extra resources, such as enough time and money to carry out interventions. Familiarization with the guidelines and planning were also time-consuming, which coincided with the findings of earlier research (Cabana et al., 1999; Lia-Hoagberg, Schaffer, & Strohschein, 1999). Structured PHNs primarily worked in organizations that had existing structures to facilitate interdisciplinary cooperation and interventions, which reduced the time needed for planning and management. Money and personnel is not enough for implementation; however, determinants like a strong organization compensate for a lack of other resources (Hoomans et al., 2007; Severens, Hoomans, Adang, & Wensing, 2013). A healthcare culture that promotes cooperation and creativity made it possible for pragmatic PHNs to structure their work and make room for implementation. These PHNs modified the guidelines to suit the resources available in their organizations. Implementation is facilitated by inventions that allow modification to suit local conditions (Greenhalgh et al., 2004). Lack of resources constituted a major barrier for critical PHNs. The municipalities in which they worked did not give priority to practice but rather downgraded it. Critical PHNs considered healthcare authorities to be uninterested in the challenges of this implementation, and they requested evaluation. Assuming the question of resources is connected to individual and organizational determinants as well as determinants concerning the innovation itself (Bahtsevani et al., 2010), if one of these barriers were to be removed, there is reason to believe that the others would be affected positively (Wandersman et al., 2008). The resigned PHNs experienced time constraints. They spent significant time on transportation and performing tasks on behalf of others, and called for guidelines that were more ready to use. Austvoll-Dahlgren and Helseth (2012) identified the lack of time as a barrier to using research. Lacking time has generally been documented to be a barrier to implementing innovations (Wandersman et al., 2008). According to Grol and Wensing (2013a), the optimal point in time at which to adjust guidelines to suit an organization is during implementation. Constructing and marketing guidelines as flexible enough to suit difference between municipalities would likely facilitate their implementation (Richter-Sundberg et al., 2015).

### Organizational embedding

The structured PHNs were part of organizations in which the guidelines were embedded at all levels. Implementation was based on local conditions, and the system allowed these PHNs to influence decision makers. Research confirms that implementation is more likely to succeed in large organizations that permit healthcare professionals to be involved in management and interdisciplinary settings (Dopson, Locock, Chambers, & Gabbay, 2001; Greenhalgh et al., 2004). Pragmatic PHNs were not initially supported by structures within their organization. However, strong leadership, a positive attitude towards the guidelines, adjusting implementation to fit the capacity, and regarding implementation as a process provided enough support to facilitate guideline implementation. Having few direct barriers to guidelines within an organization help their implementation flow more easily (Greenhalgh et al., 2004). A flexible and adaptable organization facilitates implementation of innovations (Fixsen et al., 2009). Supportive leadership has also been found to ease implementation (Bahtsevani et al., 2010). Critical PHNs were not supported by leaders’ contributions to embedding at higher organizational levels. As a result, these PHNs could not access sufficient resources and considered it unethical to implement the guidelines. Resigned PHNs found organizational embedding to be a key determinant. In addition, practitioner replacement and challenging infrastructure made implementation impossible to manage. Challenges related to administrative infrastructure have been found to be a barrier to implementation in small societies (Demby et al., 2014). An implementation strategy tailored to suit the demography would likely have facilitated implementation (Fixsen et al., 2009; Ploeg et al., 2007).

### Methodological issues

Trustworthiness in qualitative inquiry is determined by four criteria: credibility, dependability, confirmability, and transferability (Polit & Beck, 2012). The first author was familiar with the topic and conducted all interviews in an undisturbed setting at a time that suited each participant. According to the phenomenographic tradition, all questions were open-ended. Credibility was added to the data through mutual competence among all authors, rigorous discussion during analyses while bearing in mind pre-understanding, and a thorough description of the findings. Dependability refers to the stability of the data (Polit & Beck, 2012). Looking for both the predominant and non-dominant perceptions ensured that all possible ways of experiencing guideline implementation were revealed. Experiences from phenomenographic analyses have shown that 20 participants are sufficient to identify the different perceptions of phenomena (Larsson & Holmström, 2007). In this study, 18 PHNs participated but no new descriptions were identified after the eleventh interview. Those PHNs who agreed to participate had a special interest in the topic, which may have negatively affected dependability. To strengthen confirmability, all interviews were transcribed shortly after their conclusion. The first author is a PHN; the team of authors was aware of this and worked to control potential bias. Transferability or applicability to other settings was ensured using a strategic sample (Polit & Beck, 2012), namely, PHNs from all parts of Norway and from different contextual settings.

## Conclusion and implications

This study describes the various ways in which Norwegian PHNs perceive implementation of a national guideline for overweight and obesity among children and adolescents. Contextual facilitation is superior at larger organizations; however, leadership, drive, and experience compensate in smaller municipalities. At a certain point, barriers hinder implementation by exceeding the positive determinants. National guidelines are important sources of evidence for PHNs in the prevention of overweight and obesity. The diversity of contexts is challenging. These findings implicate that guideline developers should take into account the capacity for implementation of different municipalities, to increase the likelihood of success when introducing new guidelines to PHNs.
